# Hemostatic Changes Associated With Increased Mortality Rates in Hospitalized Patients With HIV-Associated Tuberculosis: A Prospective Cohort Study

**DOI:** 10.1093/infdis/jiw532

**Published:** 2016-11-07

**Authors:** Saskia Janssen, Charlotte Schutz, Amy M. Ward, Mischa A. M. Huson, Robert J. Wilkinson, Rosie Burton, Gary Maartens, Katalin A. Wilkinson, Joost C. M. Meijers, René Lutter, Martin P. Grobusch, Graeme Meintjes, Tom van der Poll

**Affiliations:** 1 Clinical Infectious Diseases Research Initiative, Institute of Infectious Disease and Molecular Medicine, University of cape Town; 2 Division of Clinical Pharmacology, Department of Medicine, University of Cape Town; 3 Department of Medicine, Groote Schuur Hospital and University of Cape Town, and; 4 Khayelitsha Hospital, Cape Town, South Africa; 5 Center of Tropical Medicine and Travel Medicine, Department of Infectious Diseases, Division of Internal Medicine; 6 Center for Experimental and Molecular Medicine, Department of Infectious Diseases, Division of Internal Medicine; 7 Department of Experimental Vascular Medicine; 8 Departments of Respiratory Medicine and Experimental Immunology, Academic Medical Center, University of Amsterdam; 9 Department of Plasma Proteins, Sanquin Research, Amsterdam, The Netherlands; 10 The Francis Crick Institute Mill Hill Laboratory, London, United Kingdom

**Keywords:** HIV, tuberculosis, coagulation, endothelium, mortality

## Abstract

**Background:**

Mortality rates remain high for human immunodeficiency virus (HIV)–associated tuberculosis, and our knowledge of contributing mechanisms is limited. We aimed to determine whether hemostatic changes in HIV-tuberculosis were associated with mortality or decreased survival time and the contribution of mycobacteremia to these effects.

**Methods:**

We conducted a prospective study in Khayelitsha, South Africa, in hospitalized HIV-infected patients with CD4 cell counts <350/µL and microbiologically proved tuberculosis. HIV-infected outpatients without tuberculosis served as controls. Plasma biomarkers reflecting activation of procoagulation and anticoagulation, fibrinolysis, endothelial cell activation, matricellular protein release, and tissue damage were measured at admission. Cox proportional hazard models were used to assess variables associated with 12-week mortality rates.

**Results:**

Of 59 patients with HIV-tuberculosis, 16 (27%) died after a median of 12 days (interquartile range, 0–24 days); 29 (64%) of the 45 not receiving anticoagulants fulfilled criteria for disseminated intravascular coagulation. Decreased survival time was associated with higher concentrations of markers of fibrinolysis, endothelial activation, matricellular protein release, and tissue damage and with decreased concentrations for markers of anticoagulation. In patients who died, coagulation factors involved in the common pathway were depleted (factor II, V, X), which corresponded to increased plasma clotting times. Mycobacteremia modestly influenced hemostatic changes without affecting mortality.

**Conclusions:**

Patients with severe HIV-tuberculosis display a hypercoagulable state and activation of the endothelium, which is associated with mortality.

Tuberculosis is the most frequent cause of hospitalization and death in human immunodeficiency virus (HIV)–infected patients worldwide [1]. Mortality rates are particularly high among HIV-infected patients who start tuberculosis treatment in the hospital, ranging from 6% to 32% [2, 3]. The reasons for this remain to be fully elucidated. Mycobacteremia is common in patients with severe HIV-associated tuberculosis (HIV-tuberculosis) [4], but its contribution to the high mortality rates is uncertain [4–6].

Bacterial sepsis is associated with activation of procoagulant responses, endothelial activation, inhibition of fibrinolysis, and decreased anticoagulant responses [7, 8]. In the most extreme cases, these changes lead to disseminated intravascular coagulation (DIC) and microvascular thrombosis [7, 9]. DIC is an important predictor of sepsis-related organ failure and death [7, 10, 11]. HIV infection can also result in hemostatic changes, with decreased concentrations of anticoagulant proteins, such as protein C and protein S [12, 13–16], and increased concentrations of coagulation and fibrinolytic markers, including D-dimer, tissue plasminogen activator (tPA), and plasminogen activator inhibitor type I (PAI-1), and markers of endothelial activation, including von Willebrand factor (vWF) and soluble vascular cell adhesion molecule 1 (VCAM-1) [12, 14, 16–19]. Although active tuberculosis has been associated with certain coagulation abnormalities [20] and increased concentrations of matricellular proteins [21, 22], much less is known about hemostatic changes during severe HIV-tuberculosis. Moreover, data on the impact of mycobacteremia on coagulation abnormalities in HIV-infected patients are, to the best of our knowledge, not available.

We hypothesized that HIV-tuberculosis is accompanied by hemostatic changes that resemble those found in bacterial sepsis, especially in patients with mycobacteremia, and that these changes would be associated with mortality.

In the current study we aimed to determine whether hemostatic changes, including coagulation, matricellular protein concentrations and endothelial activation in hospitalized patients with HIV-tuberculosis are associated with 12-week mortality rates. We also aimed to describe differences in concentrations of these markers in patients with HIV-tuberculosis with or without mycobacteremia, and in HIV-infected controls without active tuberculosis.

## MATERIALS AND METHODS

### Study Design and Population

This prospective, observational cohort study was conducted in Khayelitsha, Cape Town, South Africa. This township has a reported antenatal HIV seroprevalence of 34% [23] and a tuberculosis notification rate of 917 per 100 000 persons (City of Cape Town, unpublished 2015 data). Patients were recruited at 2 sites: Khayelitsha Hospital, a public sector hospital in the township, and the Ubuntu Clinic, an outpatient clinic in Khayelitsha.

**Figure 1.**
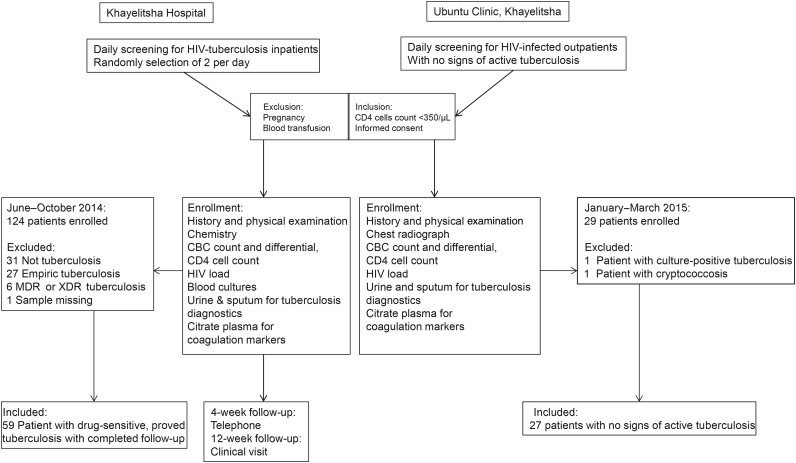
Study flow. Flow diagram showing an overview of methods, patient recruitment, and criteria for inclusion in this study. Between June and November 2014, we recruited adult patients immunodeficiency virus (HIV) infection, CD4 cell counts <350/µL, and a tuberculosis diagnosis or a high clinical suspicion of tuberculosis on admission to Khayelitsha Hospital. Patients who were pregnant or received a blood transfusion were excluded, and only patients with microbiologically proved rifampicin-susceptible tuberculosis were included in the analyses. A random selection procedure was used to select 2 eligible patients on each weekday. As controls, HIV-infected outpatients with CD4 cell counts <350/µL but active tuberculosis were recruited at the Ubuntu Clinic in Khayelitsha. Abbreviations: CBC, complete blood cell; MDR, multidrug resistant; XDR, extensively drug resistant.

The study populations are summarized in Figure 1. Nonpregnant HIV-infected patients with CD4 cell counts <350/µL, with newly diagnosed tuberculosis or a high clinical suspicion of tuberculosis on admission to Khayelitsha Hospital were recruited between June and October 2014. Only those patients with microbiologically proved rifampicin-susceptible tuberculosis were included in the analyses reported here. Selection bias was minimized by using a random selection procedure. During weekdays the emergency and medical wards were screened for patients fulfilling inclusion criteria. Two patients a day were selected through a randomization process using dice. Nonpregnant HIV-infected outpatients with CD4 cell counts <350/µL without active tuberculosis were recruited at the Ubuntu Clinic as HIV-infected control patients.

### Ethics

Ethical approval was obtained from the University of Cape Town Faculty of Health Sciences Human Research Ethics Committee (reference Nos. 057/2013 and 568/2014). Written informed consent was sought from all patients. Patients who were initially too ill to provide consent were enrolled and monitored daily. Patients were invited to provide informed consent or withdraw from the study once they regained capacity to consent. If a patient died before consent was obtained, permission to include his or her data was obtained from the University of Cape Town Faculty of Health Sciences Human Research Ethics Committee.

### Procedures

Samples were obtained before initiation of tuberculosis treatment in all patients with HIV-tuberculosis included. Sputum (spontaneous or induced), when produced, was sent for tuberculosis culture and the Xpert MTB/RIF assay (Cepheid). The Xpert MTB/RIF test for urine was performed on concentrated urine samples. MycoFLytic blood cultures (Becton Dickinson Biosciences) for tuberculosis were inoculated with 5 mL of whole blood and cultured for 42 days for patients with HIV-tuberculosis. Mycobacteremia was defined as *Mycobacterium tuberculosis* growing in ≥1 MycoFlytic blood culture, confirmed by GenoType MTBDRplus assay (Hain Lifescience). Drug sensitivity testing for isoniazid and rifampicin was done on all positive cultures*.* Full blood counts and differentials (Roche XN-10 Sysmex), HIV loads (Abbott M2000 SP/RT) and CD4 cell counts (Beckman Coulter FC 500 Analysis Cellmek Preparation) were performed at the National Health Laboratory Service laboratory. Patients with HIV-tuberculosis were followed up by telephone at 4 weeks and clinical review at 12 weeks. If contact could not be established, regional clinical laboratory and pharmacy systems were used to ascertain vital status at 12 weeks. Controls were screened for active tuberculosis with a tuberculosis symptom screen [24], sputum culture, and urine and sputum Xpert MTB/RIF assays. They were excluded if results of symptom screen for tuberculosis or any tuberculosis diagnostic tests were positive.

### Data Sources and Measurement

#### Clinical Data

Clinical data were captured from patient files, history and physical examination. Results of study-specific and other relevant tests were captured from the online National Health Laboratory Service database [25].

#### Plasma Processing

Citrated whole blood was obtained from patients with HIV-tuberculosis and controls and kept at 4°C. Plasma was extracted within 3 hours before being stored at −80°C until further analyses, which were performed at the Academic Medical Center in Amsterdam. Premixed multiplex assays (R&D Systems), performed with a Bioplex 200 system (Bio-Rad), were used to measure concentrations of D-dimer, tPA (both eBioscience), PAI-1, platelet factor 4 (PF-4), protein C, angiopoietin 1 and 2 (Ang-1 and Ang-2), tenascin C, metallopeptidase inhibitor 1 (TIMP-1), cardiac troponin I (cTNI), trefoil factor 3 (TFF3), cystatin C, neutrophil gelatinase-associated lipocalin (NGAL), soluble VCAM-1, soluble E-selectin, and soluble tyrosine kinase with immunoglobulinlike and epidermal growth factor–like domains 1 and 2 (Tie-1 and Tie-2).

Prothrombin time (PT), activated partial thromboplastin time (aPTT), and concentrations of coagulation factors II, V, VII, VIII, IX, X and XI and antithrombin were measured using an automated blood coagulation analyzer (BCS XP, Siemens Healthcare Diagnostics). The fibrinogen concentration was derived from the change in optical signal in PT; the international normalized ratio (INR) was calculated using the mean PT provided by the manufacturer. Protein S (total) and vWF concentrations were measured with in-house assays containing antibodies (Dako). Free protein S was measured by precipitating the C4b-binding protein-bound fraction with polyethylene glycol 8000 and measuring the concentration of free protein S in the supernatant. The activity of a disintegrin and metalloproteinase with a thrombospondin type 1 motif, member 13 (ADAMTS-13) was assessed as described elsewhere [26], with a BCS-XP automated coagulation instrument (Siemens). Factor I + II (FI + II) was measured using Enzygnost F1 + 2 (Siemens). The DIC score (International Society for Thrombosis and Haemostasis) was calculated based on platelet counts, plasma D-dimer and fibrinogen levels, and PT prolongation, as described elsewhere (Supplementary Table) [9].

#### Statistical Analysis

Data were analyzed using SPSS Version 22 (IBM), GraphPad PRISM version 6, and R statistical manager. Categorical variables are presented as percentages, and continuous variables as medians with interquartile ranges. We used χ² or Fisher exact tests for categorical data, 1-way analysis of variance and Student *t* test for parametric continuous data, and Kruskal–Wallis and Mann–Whitney *U* tests for nonparametric data. Variables were investigated for their association with time to death in a Cox proportional hazard model, adjusted for confounders. A priori–defined potential confounders were age, sex, antiretroviral therapy status, HIV load and CD4 cell count. A potential confounder was retained in the final model if introduction of the confounder to the model lead to a >10% change of the effect measure.

In a secondary analyses the influence of tuberculosis and mycobacteremia on alteration of biomarkers was assessed. Variables were compared for HIV-infected controls versus patients with HIV-tuberculosis, and for patients with HIV-tuberculosis who had mycobacteremia versus those who did not. Patients receiving treatment with prophylactic or therapeutic anticoagulants (warfarin, heparin, or enoxaparin; n = 14) were excluded from analyses involving clotting times, fibrinogen, and coagulation factors (including DIC). All reported *Q* values were calculated using Benjamini-Hochberg procedures for multiple-testing correction [27]; differences were considered significant at *P* < .05 and *Q* < 0.10 were regarded as significant.

## RESULTS

### Patients

Of 124 HIV-infected patients with probable tuberculosis enrolled, 59 patients with confirmed rifampicin-susceptible tuberculosis were included in this analysis. Twenty-seven HIV-infected control patients with CD4 cell counts <350/µL and negative results of screening for tuberculosis were included (Figure 1).

Table 1 displays baseline clinical data for the study cohort. Compared with HIV-infected controls, patients with HIV-tuberculosis were more often anemic and had higher white blood cell counts. At 12-week follow-up, 16 patients with HIV-tuberculosis (27%) had died, at a median of 12 days (interquartile range, 0–24 days) after enrollment; no patients were lost to follow-up.

**Table 1.** Clinical Characteristics and Hematological Parameters^a^

Characteristic or Parameter	HIV-Infected Controls (n = 27)	Patients With HIV-Associated Tuberculosis (n = 59)	*Q* Value^b^	Survivors (n = 43)	Patients Who Died (n = 16)	*Q* Value^c^
Demographic characteristics						
Male sex, No. (%)	8/27 (30)	29/59 (49)	0.30	20/43 (47)	6/16 (38)	0.77
Age, median (IQR), y	34 (29–46)	39 (33–45)	0.35	36 (31–41)	44 (35–55)	0.22
ART status, No. (%)			0.95			0.23
Naive	18/27 (67)	33/59 (56)		22/43 (51)	6/16 (38)	
On ART	2/27 (11)	13/59 (22)		9/43 (21)	5/16 (31)	
Defaulted	6/27 (22)	13/59 (22)		12/43 (28)	5/16 (31)	
Anticoagulant use at enrolment, No. (%)						
Prophylactic	0/27 (0)	13/59 (22)	0.01^d^	9/43 (21)	4/16 (25)	0.83
Therapeutic	0/27 (0)	1/59 (2)	1.00	1/43 (2)	0/16 (0)	1.00
HIV disease markers, median (IQR)						
CD4 cell count, cells/µL	144 (67–204)	72 (35–168)	0.35	70 (35–159)	45 (19–90)	0.83
HIV load, log copies/mL	4.28 (1.59–4.91)	5.03 (3.06–5.85)	0.47	5.03 (3.06–5.83)	4.39 (3.18–5.69)	0.91
Tuberculosis diagnostics, No. (%)						
Sputum culture/Xpert MTB/RIF positive^e^	0/22 (0)	43/50 (86)	ND	34/40 (85)	9/10 (90)	0.83
Urine Xpert MTB/RIF positive	0/27 (0)	20/54 (37)	ND	14/43 (33)	6/9 (66)	0.23
Mycobacteremia	ND	30/59 (51)	ND	23/43 (54)	7/16 (44)	0.77
Hematological parameters, median (IQR)						
Hemoglobin, g/dL	12.1 (10.8–12.8)	8.9 (6.7–10.8)	0.002^d^	9.0 (6.9–11.2)	6.9 (6.4–9.8)	0.23
Blood cell count, × 10^9^/L						
WBCs	4.3 (3.5–5.8)	6.67 (4.48–9.52)	0.002^d^	7.1 (4.8–10.4)	5.8 (3.9–7.6)	0.23
Neutrophils	2.2 (1.5–3.1)	5.82 (3.79–9.30)	0.002^d^	6.1 (4.3–9.5)	4.7 (2.7–6.8)	0.23
Lymphocytes	1.53 (0.96–1.96)	0.56 (0.36–0.96)	0.002^d^	0.60 (0.38–1.03)	0.49 (0.29–0.78)	0.32
Monocytes	0.36 (0.32–0.43)	0.34 (0.17–0.52)	0.49	0.39 (0.20–0.58)	0.20 (0.12–0.43)	0.23

Abbreviations: ART, antiretroviral therapy; HIV, human immunodeficiency virus; IQR, interquartile range; ND, not done; WBCs, white blood cells.

^a^ For comparisons, χ² and Fisher exact tests were used for categorical data, and Mann–Whitney *U* tests for continuous data.

^b^*Q* values for comparisons between HIV-infected control groups and patients with HIV-associated tuberculosis.

^c^*Q* values for comparisons between patients with HIV-associated tuberculosis who died and survived.

^d^ Significant at *Q* < 0.10.

^e^ Five HIV-infected control patients and 9 patients with HIV-tuberculosis were unable to produce sputum.

Mycobacteremia was detected in 30 of 59 patients (51%) with HIV-tuberculosis. Rates at 12 weeks did not differ between patients with HIV-tuberculosis with or without mycobacteremia, and mycobacteremia was not associated with decreased survival time (crude hazard ratio, 0.77 [95% confidence interval, .29–2.06; *P* = .60]; adjusted hazard ratio, 0.84; [.30–2.38; *P* = .75]).

### Activation of the Coagulation System

**Figure 2.**
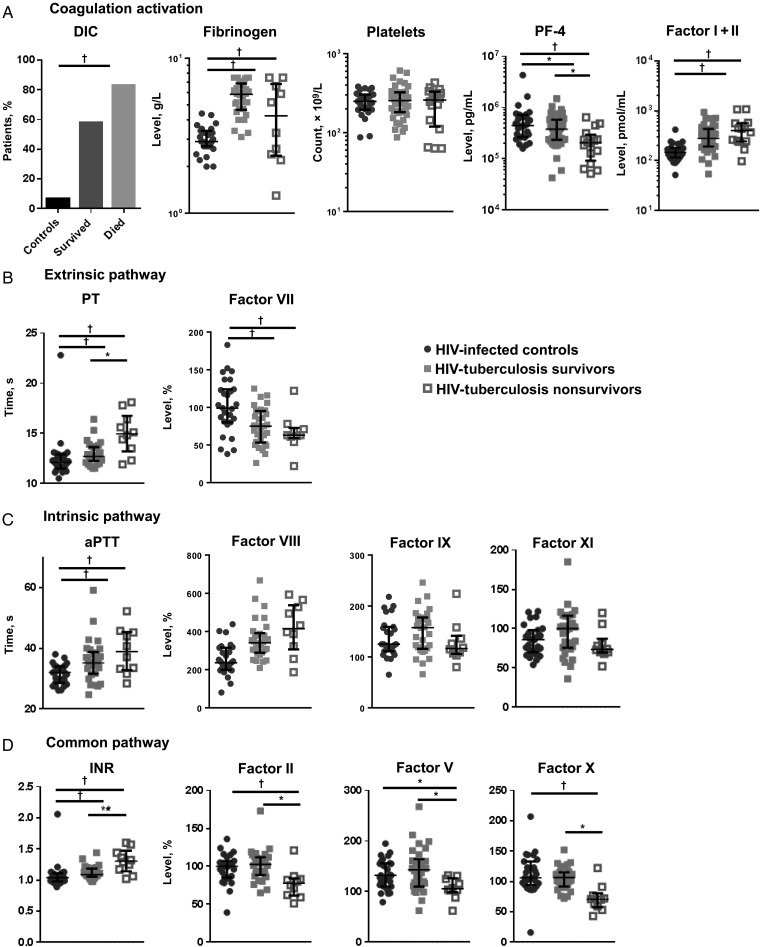
Coagulation activation and clotting times. Figure showing the percentage of patients fulfilling criteria for disseminated intravascular coagulation (DIC). Median concentrations and interquartile ranges are shown for markers of coagulation and platelet activation (platelet factor 4 [PF-4]) (*A*), clotting times (prothrombin time [PT], activated partial thromboplastin time [aPTT], and international normalized ratio [INR]), and concentrations of coagulation factors involved in the extrinsic (*B*), intrinsic (*C*), and common (*D*) coagulation pathways. Values are shown for human immunodeficiency virus (HIV)–infected controls (*blackcircles*), patients with HIV-associated tuberculosis (HIV-tuberculosis) who survived (*gray squares*), patients with HIV-tuberculosis who died (*open squares*). Kruskal–Wallis and Mann–Whitney *U* tests were used for comparisons between groups. HIV-infected controls were compared with patients with HIV-tuberculosis, and patients with HIV-tuberculosis who survived were compared with those who died. Levels of significance: *Q < 0.05; ^†^*Q* < 0.01.

Coagulation activation can lead to consumption and depletion of coagulation factors. In the comparison between HIV-infected controls and patients with severe HIV-tuberculosis, marked differences were seen in markers of coagulation. PT, aPTT, and INR concentrations were all more increased in patients with HIV-tuberculosis, and concentrations of factor VII were significantly lower (Figure 2). Fibrinogen and FI + II concentrations were higher in patients with HIV-tuberculosis than in controls, and PF-4 concentrations were lower (Figure 2).

**Figure 3.**
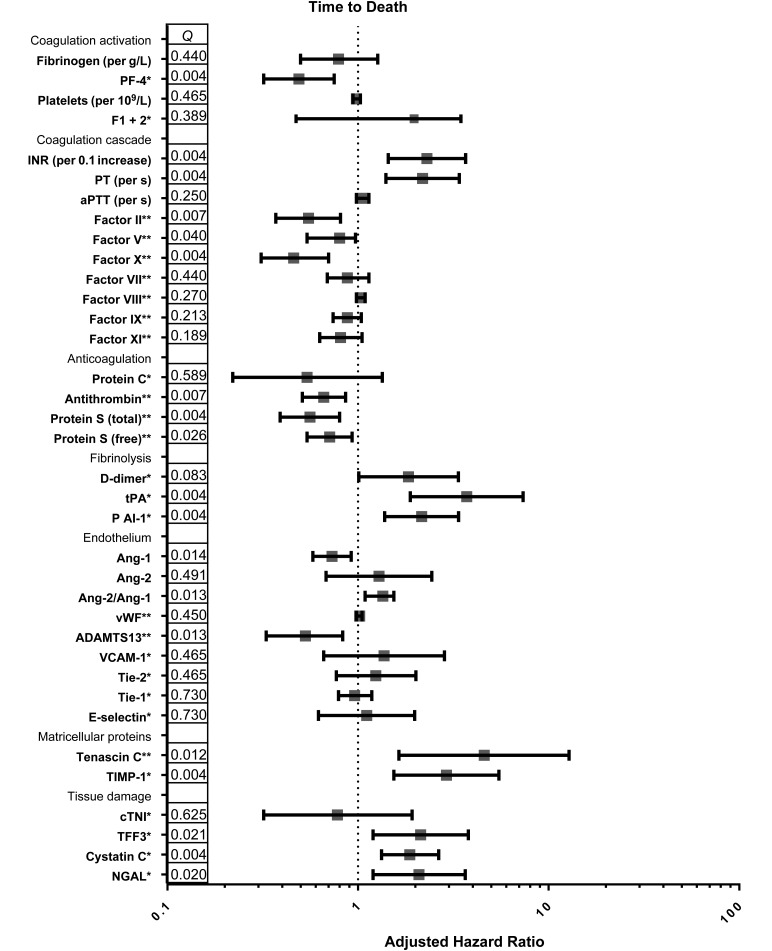
Hemostatic changes and time to death. Figure shows adjusted hazard ratios and 95% confidence intervals for respective variables, calculated with Cox proportional hazard analysis. A priori–defined potential confounders were age, sex, antiretroviral therapy status, human immunodeficiency virus (HIV) load and CD4 cell count. A potential confounder was retained in the final model if introduction of the confounder to the model lead to a >10% change of the effect measure. *Q* values (corrected *P* values) are shown per log_2_ pg/mL increase (*) or per 10% increase (**), depending on assay used. Abbreviations: ADAMTS-13, a disintegrin and metalloproteinase with a thrombospondin type 1 motif, member 13; Ang 1 and Ang 2, angiopoietin 1 and 2; aPTT, activated partial thromboplastin time; cTNI, cardiac troponin I; FI + II, factor I + II; INR, international normalized ratio; NGAL, neutrophil gelatinase-associated lipocalin; PAI-1, plasminogen activator inhibitor type I; PF-4, platelet factor 4; PT, prothrombin time; TFF3, trefoil factor 3; Tie-1 and Tie-2 tyrosine kinase with immunoglobulinlike and epidermal growth factor–like domains 1 and 2; TIMP-1, metallopeptidase inhibitor 1; tPA, tissue plasminogen activator; VCAM-1, vascular cell adhesion protein 1; vWF, von Willebrand factor.

In patients with HIV-tuberculosis who died, fibrinogen concentrations and platelet counts were similar to those in patients who survived, but FI + II concentrations were slightly higher. Lower concentrations of PF-4 were associated with decreased survival time (Figures 2 and 3). Coagulation factors of the common pathway were most profoundly depleted in patients with HIV-tuberculosis who died, in contrast to the intrinsic pathway factors, whose concentrations were similar between patients who died and those who survived (Figure 2). PT and INR were more prolonged in patients who died, but there was no difference in aPTT. Lower concentrations of common pathway factors II, V, and X were independently associated with decreased survival time, in addition to prolonged PT and higher INR (Figure 3).

DIC was common among patients with HIV-tuberculosis; 19 of 33 survivors (58%) and 10 of 12 nonsurvivors (83%) fulfilled criteria for DIC at presentation (Figure 2). There was no statistically significant association between DIC and decreased survival time (crude hazard ratio, 0.32 [95% confidence interval, .07–1.48; *P* = .15]; adjusted hazard ratio, 0.46 [09–2.32; *P* = .35]).

Among patients with HIV-tuberculosis, those with mycobacteremia had lower platelet counts than those with negative blood cultures. No further differences were seen in coagulation markers and clotting times between patients with and those without mycobacteremia (Table 2).

**Table 2.** Biomarker Concentrations and Mycobacteremia

Biomarker	Median (IQR)		
	Nonmycobacteremic Patients (n = 29)	Mycobacteremic Patients (n = 30)	*Q* Value^a^
Coagulation activation			
Fibrinogen, g/L	5.2 (3.4–6.2)	6.3 (4.5–7.5)	0.52
PF-4, µg/mL	0.31 (0.21–0.48)	0.32 (0.18–0.48)	0.56
Platelets, × 10^9^/L	311 (234–380)	202 (124–276)	0.04^b^
PT, s	12.5 (12.1–13.7)	13.6 (12.6–15.4)	0.22
aPTT, s	34.2 (31.3–37.1)	39.5 (34.8–42.6)	0.13
INR, s	1.04 (1.07–1.19)	1.08 (1.18–1.34)	0.22
Factor, %			
II	101 (83–112)	89 (78–108)	0.56
V	128 (103–160)	128 (105–155)	0.89
VII	71 (62–95)	64 (52–88)	0.73
VIII	378 (302–494)	332 (288–393)	0.22
IX	158 (117–185)	119 (109–162)	0.22
X	106 (91–112)	90 (72–113)	0.52
XI	100 (78–117)	76 (65–107)	0.22
Anticoagulant proteins			
Antithrombin, %	90 (69–103)	85 (75–96)	0.75
Protein S (total), %	88 (76–99)	94 (76–109)	0.56
Protein S (free), %	54 (39–66)	51 (40–73)	0.71
Protein C, µg/mL	0.40 (0.34–0.60)	0.37 (0.29–0.43)	0.22
Fibrinolysis			
D-dimer, µg/mL	9.3 (7.0–16.6)	10.4 (7.1–16.6)	0.68
tPA, ng/mL	97 (53–161)	140 (112–234)	0.08^b^
PAI-1, ng/mL	28 (18–34)	45 (22–69)	0.09^b^
Endothelial activation			
E-selectin, µg/mL	0.10 (0.06–0.13)	0.12 (0.08–0.15)	0.46
vWF, %	471 (406–593)	608 (519–696)	0.08^b^
ADAMTS-13, %	41 (26–57)	29 (20–43)	0.09^b^
VCAM-1, µg/mL	3.91 (2.27–6.63)	6.42 (4.76–7.78)	0.08^b^
Ang-1, ng/mL	1.3 (0.8–1.6)	6.2 (0.3–1.3)	0.09^b^
Ang-2, ng/mL	9.5 (5.5–15.4)	8.2 (5.4–10.4)	0.58
Ang-2/Ang-1 ratio	6.4 (3.7–15.3)	13.8 (5.0–29.6)	0.22
Tie-2, pg/mL	396 (235–763)	362 (227–517)	0.58
Tie-1, ng/mL	30 (12–39)	16 (0.7–25)	0.08^b^
Matricellular proteins			
Tenascin C, ng/mL	38 (27–49)	35 (28–46)	0.73
TIMP-1, µg/mL	0.29 (0.16–0.43)	0.28 (0.18–0.41)	0.77
Tissue damage			
cTNI, pg/mL	338 (303–444)	317 (254–398)	0.39
TFF3, pg/mL	5120 (3682–7747)	4889 (2756–10 951)	0.73
Cystatin C, µg/mL	0.81 (0.70–1.20)	1.07 (0.73–1.97)	0.39
NGAL, µg/mL	0.15 (0.09–0.29)	0.19 (0.09–0.25)	0.64

Abbreviations: ADAMTS-13, a disintegrin and metalloproteinase with a thrombospondin type 1 motif, member 13; Ang-1 and Ang-2, angiopoietin 1 and 2; aPTT, activated partial thromboplastin time; cTNI, cardiac troponin I; INR, international normalized ratio; IQR, interquartile range; NGAL, neutrophil gelatinase-associated lipocalin; PAI-1, plasminogen activator inhibitor type I; PF-4, platelet factor 4; PT, prothrombin time; TFF3, trefoil factor 3; Tie-1 and Tie-2, tyrosine kinase with immunoglobulinlike and epidermal growth factor–like domains 1 and 2; TIMP-1, metallopeptidase inhibitor 1; tPA, tissue plasminogen activator; VCAM-1, vascular cell adhesion protein 1; vWF, von Willebrand factor.

^a^*Q* values are shown for Mann–Whitney *U* tests for comparisons between patients with and patients without mycobacteremia.

^b^ Significant at *Q* < 0.10.

### Anticoagulant Proteins

**Figure 4.**
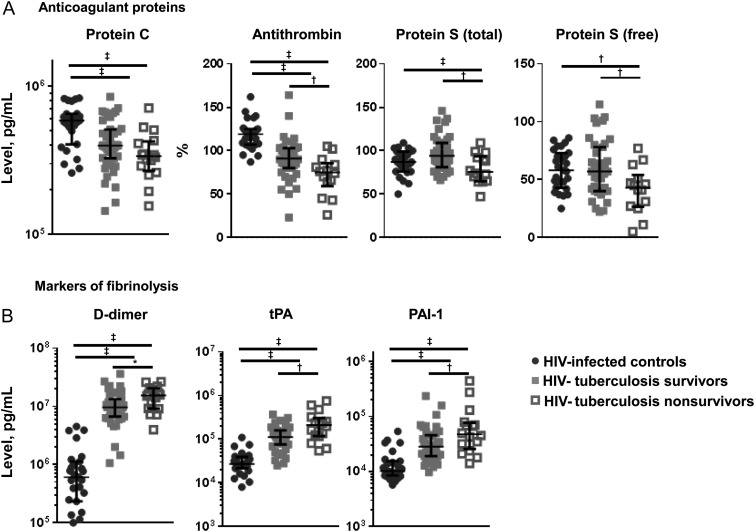
Anticoagulation and fibrinolysis. Figure shows median concentrations and interquartile ranges for anticoagulant protein C, protein S (total and free) and antithrombin (*A*) and for markers of fibrinolysis D-dimer, tissue plasminogen activator (tPA) and plasminogen activator inhibitor type 1 (PAI-1) (*B*). Values are shown for human immunodeficiency virus (HIV)–infected controls (*black circles*), patients with HIV-associated tuberculosis who survived (*gray squares*), and patients with HIV-tuberculosis who deceased (*open squares*). Kruskal–Wallis and Mann–Whitney *U* tests were used for comparisons between groups. HIV-infected controls were compared with patients with HIV-tuberculosis, and patients with HIV-tuberculosis who survived were compared with those who died. Levels of significance: *Q < 0.10; ^†^*Q* < 0.05; ^‡^*Q* < 0.01.

Concentrations of anticoagulant proteins antithrombin and protein C were reduced in patients with HIV-tuberculosis compared with HIV-infected controls, indicating decreased anticoagulation in active tuberculosis (Figure 4*A*). These differences were more pronounced in patients with HIV-tuberculosis who died; concentrations of anticoagulant molecules antithrombin and protein S (total and free) were reduced relative to those in patients with HIV-tuberculosis who survived (Figure 4*A*). Lower concentrations of these molecules were independently associated with decreased survival time (Figure 3). No differences in concentrations of anticoagulant proteins were seen between patients with and those without mycobacteremia (Table 2).

### Fibrinolytic Response

Markers of fibrinolysis, D-dimer, tPA, and PAI-1, were increased in patients with HIV-tuberculosis compared with HIV-infected controls (Figure 4*B*). Patients who died had higher concentrations of markers of fibrinolysis than those who survived (Figure 4*B*). Higher concentrations of D-dimer, tPA, and PAI-1 were independently associated with decreased survival time (Figure 3). Concentrations of tPA and PAI-1 were higher in mycobacteremic patients than in patients with HIV-tuberculosis without mycobacteremia (Table 2), but there were no differences in D-dimer concentrations.

### Activation of the Vascular Endothelium

**Figure 5.**
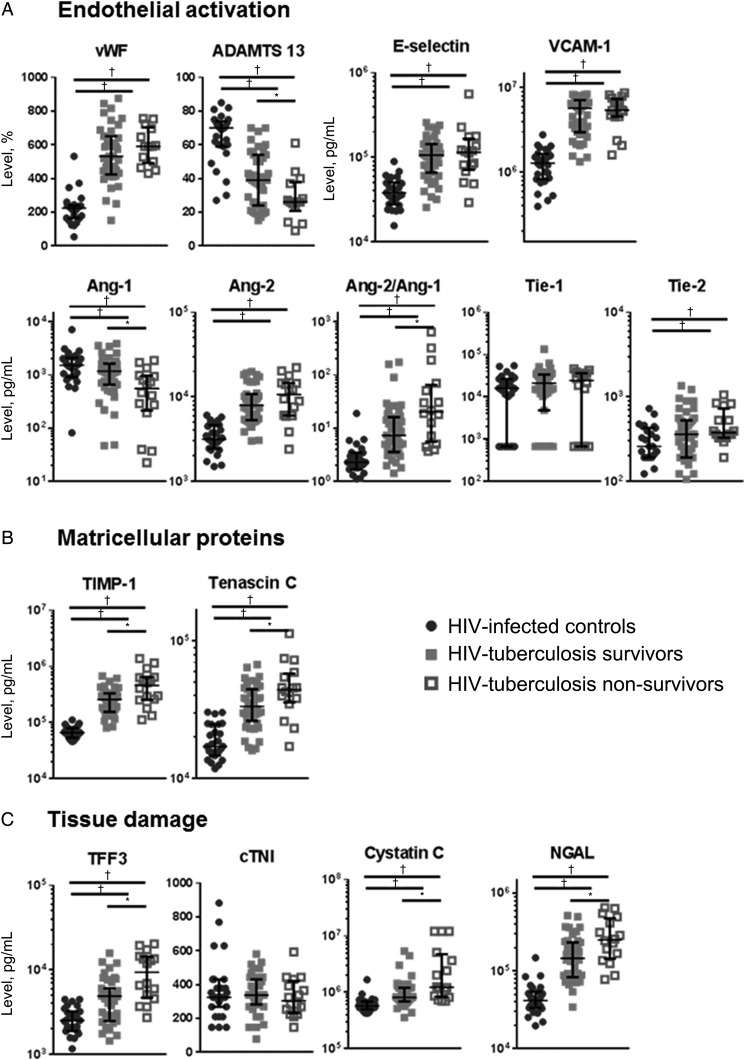
Endothelial activation, extracellular matrix molecules and tissue damage. Figure shows median concentrations and interquartile ranges for markers of endothelial activation (von Willebrand factor [vWF], a disintegrin and metalloproteinase with a thrombospondin type 1 motif, member 13 [ADAMTS-13], E-selectin, soluble vascular cell adhesion protein 1 [VCAM-1], angiopoietin 1 and 2 [Ang-1 and Ang-2], and Ang-1/Ang-2 ratio) (*A*), matricellular proteins (tenascin C and metallopeptidase inhibitor 1 [TIMP-1]) (*B*), and markers of tissue damage (cardiac troponin I [cTNI], cystatin C, neutrophil gelatinase-associated lipocalin [NGAL], and trefoil factor 3 [TFF3]) (*C*). Values are shown for human immunodeficiency virus (HIV)–infected controls (*black circles*), patients with HIV-associated tuberculosis who survived (*gray squares*), and patients with HIV-tuberculosis who deceased (*open squares*), respectively. Kruskal–Wallis and Mann–Whitney *U* tests were used for comparisons between groups. HIV-infected controls were compared with patients with HIV-tuberculosis, and patients with HIV-tuberculosis who survived were compared with those who died. Levels of significance: *Q < 0.05; ^†^*Q* < 0.01.

In patients with HIV-tuberculosis, concentrations of all markers of endothelial activation measured were altered compared with HIV-infected controls, including higher concentrations of vWF, E-selectin, VCAM-1, Ang-2 and Tie-2 and lower concentrations of ADAMTS-13 and Ang-1, leading to an increased Ang-2/Ang-1 ratio in patients with HIV-tuberculosis (Figure 5). Concentrations of tenascin C and TIMP-1, both markers of matricellular metabolism, were higher in patients with HIV-tuberculosis (Figure 5).

ADAMTS-13 concentrations were lower in patients with HIV-tuberculosis who died than in survivors, possibly owing to higher concentrations of vWF (Figure 5). Lower concentrations of ADAMTS-13 were associated with decreased survival time (Figure 3). In addition, Ang-1 concentrations were lower with higher concentrations of Ang-2, leading to an increased Ang-2/Ang-1 ratio in those who died (Figure 5). Low Ang-1 concentrations and high Ang-2/Ang-1 ratios were independently associated with mortality (Figure 3). There were no differences in concentrations of VCAM-1, E-selectin, Tie-1 and Tie-2 expression between patients with HIV-tuberculosis who survived and those who died.

Higher concentrations of matricellular proteins tenascin C and TIMP-1 were independently associated with decreased survival time (Figure 5*B* and 3). Concentrations of vWF and VCAM-1 were higher in mycobacteremic patients than in patients with HIV-tuberculosis without mycobacteremia, whereas concentrations of ADAMTS-13, Ang-1, and Tie-1 were lower (Table 2). There were no differences in concentrations of markers of matricellular homeostasis between mycobacteremic patients and patients with HIV-tuberculosis without mycobacteremia (Table 2).

### Tissue Injury Markers

The hemostatic changes described above may lead to microvascular thrombosis and organ damage. Markers of intestinal damage (TFF3) and kidney injury (cystatin C and NGAL) were increased in patients with HIV-tuberculosis compared with HIV-infected controls (Figure 5*C*). There were no differences in cTNI, a marker of cardiac tissue damage.

Patients with HIV-tuberculosis who died presented with higher concentrations of TFF3, cystatin C, and NGAL (Figure 5*C*). Higher concentrations of these molecules were independently associated with decreased survival time (Figure 3). Concentrations of cTNI did not differ between these groups. There were no differences in concentrations of markers of tissue damage between mycobacteremic patients with HIV-tuberculosis and patients without mycobacteremia (Table 2).

## DISCUSSION

We report a procoagulant state in HIV-associated patients with tuberculosis, together with signs of endothelial activation and tissue damage, which was associated with higher mortality rates. Specifically, our main findings are that concentrations for markers of fibrinolysis (D-dimer, tPA, and PAI-1), endothelial activation (Ang-2, Ang-2/Ang-1 ratio, and vWF), extracellular matrix metabolism (tenascin C and TIMP-1) and tissue damage (TFF3, cystatin C, and NGAL) were increased in patients with HIV-tuberculosis who died, whereas concentrations of anticoagulant proteins (total and free protein S, ADAMTS-13 and antithrombin) were decreased. Alterations in the majority of these markers were independently associated with decreased survival time in patients with HIV-tuberculosis. Coagulation activation results in depletion of coagulation factors; this was seen especially for factors involved in the common pathway (factor II, V and X) and lower concentrations of all of these were associated with decreased survival time. Consequently, clotting times (PT and aPTT) were prolonged in patients who died. These changes are likely to be driven by tuberculosis rather than advanced HIV only, as suggested by the more marked alterations of these markers observed in patients with HIV-tuberculosis, compared with HIV-infected controls.

Our study provides an integrated overview of markers of coagulation, endothelial activation, fibrinolysis, extracellular matrix metabolism, and tissue damage alterations in HIV-tuberculosis. The early mortality rates [2, 3] and prevalence of mycobacteremia [5, 28, 29] reported here are similar to findings of other studies in Africa, securing the external validity of our study. Ethics committee permission was obtained to recruit drowsy or confused patients with deferred consent, as explained in Materials and Methods. Inclusion of this patient category increases the generalizability of our results to the most critically ill patients, because patients with severe HIV-tuberculosis are frequently confused or drowsy at the time of hospital admission owing to neurological tuberculosis or the severity of their disease.

Decreased concentrations of protein C were associated with tuberculosis in HIV-infected patients, whereas reduced protein S concentrations (deficits in concentrations of both total and free protein S) were associated with decreased survival time. Under physiological conditions, approximately 60% of protein S is bound to the β chain of C4 binding protein; only the free form has protein C cofactor activity [30]. Concentrations of C4 binding protein increase in inflammatory conditions, leading to reduced levels of free (active) protein S [31]. Because protein S is produced predominantly in the liver and endothelium, reduced free protein S may result from liver failure or endothelial dysfunction [32] and/or increased consumption. A previous study showed that HIV-infected patients with bacterial sepsis had more profound deficiencies of free protein S [33], but that study did not assess associations with death.

Inflammation leads to endothelial activation, inducing release of vWF, which enhances platelet binding and thereby clot formation [34]. Under normal conditions, vWF is regulated by ADAMTS-13 [35]. Ang-1 and Ang-2 are both ligands for the Tie-2 receptor. In healthy individuals, Ang-1 concentrations exceed Ang-2 concentrations, triggering prosurvival pathways and inhibiting proinflammatory responses [8]. These markers of endothelial activation were increased in HIV-tuberculosis, and more profoundly so in patients who died. Previous studies have reported endothelial activation in tuberculosis [20, 36, 37] as well as in HIV infection [38, 12, 16–19]. One study in HIV-infected women in Kenya showed increased concentrations of Ang-2 at baseline, which decreased with antiretroviral therapy [14]. However, the literature on endothelial activation in HIV-tuberculosis coinfection is scarce. Increased concentrations of Ang-2 indicate a role for endothelial dysfunction in HIV-tuberculosis, possibly contributing to mortality. This might be explained by endothelial dysfunction-related vascular leakage, leading to impaired tissue oxygenation and organ damage [8]. Currently, therapeutic agents targeting the angiopoietin/Tie-2 system and Ang-2 antagonists are being developed and evaluated in cancer treatment [39–41]. Our findings suggest it may be worthwhile investigating these agents as a host-directed adjunctive therapy for severe HIV-tuberculosis.

Imbalances in extracellular matrix homeostasis have been described in active tuberculosis, and proteolytic activity of matrix metalloproteinases have been related to dissemination of *Mycobacterium tuberculosis* [21]. Concentrations of matricellular proteins tenascin C and TIMP-1 were increased in patients with HIV-tuberculosis, and higher concentrations were associated with decreased survival time. This is in agreement with findings of previous studies that have demonstrated increased levels of tenascin C and TIMP-1 in patients with active tuberculosis [42], with even higher concentrations measured at the site of infection [43]. Matrix metalloproteinase inhibitors have been investigated as cancer treatment [44] and have been suggested for host-directed therapy in tuberculosis [45]. It remains to be established whether these agents may be of value in HIV-tuberculosis therapy.

Concentrations of NGAL and cystatin C, both early markers of renal injury, were increased in patients with HIV-tuberculosis and associated with decreased survival time. This suggests renal injury in severe HIV-associated tuberculosis. Likewise, TFF3, a marker of intestinal damage, was elevated in patients with HIV-tuberculosis and associated with decreased survival time. Bacterial product translocation due to dysfunction of the intestinal barrier has been widely described in HIV-infection [46–48] and has been reported in tuberculosis as well [49, 50].

Mycobacteremia was not associated with decreased survival time and did not affect markers of coagulation, anticoagulation, extracellular matrix or tissue damage. Markers of fibrinolysis (tPA and PAI-1) had increased concentrations in mycobacteremia, as did markers of endothelial activation VCAM-1 and vWF, whereas concentrations of Ang-1 and ADAMTS-13 were decreased. Mortality rates were high and DIC was frequent in our cohort, regardless of mycobacteremia, suggesting a limited prognostic value of mycobacterial blood cultures in patients with severe HIV-tuberculosis.

Our study has limitations. We do not have data on the cause of death in patients who died. Owing to logistic and sociocultural issues (none of the families gave their consent), postmortem examinations were not performed. Our findings cannot be related to clinical outcomes other than death.

Patients with severe HIV-tuberculosis display a hypercoagulable state that is associated with mortality. DIC is a common finding in this patient category. Our data indicate that activation of coagulation and the endothelium, together with decreased activity of anticoagulant pathways, were associated with depletion of coagulation factors and increased clotting times. This may lead to thromboembolic events, microvascular thrombosis, and tissue damage, as illustrated by increased concentrations for markers of tissue damage. This study is the first to investigate coagulation abnormalities in HIV-tuberculosis and suggests that treatment strategies targeting hemostasis, endothelial activation, and matricellular homeostasis may be of interest for evaluation in patients with severe HIV-tuberculosis who continue to have an unacceptably high mortality rate.

## Supplementary Material

Supplementary Data

## References

[1] Ford N, Shubber Z, Meintjes G et al Causes of hospital admission among people living with HIV worldwide: a systematic review and meta-analysis. Lancet HIV 2015; 2:e438–44. 26423651 10.1016/S2352-3018(15)00137-X

[2] Kyeyune R, den Boon S, Cattamanchi A et al Causes of early mortality in HIV-infected TB suspects in an East African referral hospital. J Acquir Immune Defic Syndr 2010; 55:446–50. 21105258 10.1097/qai.0b013e3181eb611a PMC3249444

[3] Odone A, Amadasi S, White RG, Cohen T, Grant AD, Houben RM The impact of antiretroviral therapy on mortality in HIV positive people during tuberculosis treatment: a systematic review and meta-analysis. PLoS One 2014; 9:e112017. 25391135 10.1371/journal.pone.0112017 PMC4229142

[4] Crump JA, Ramadhani HO, Morrissey AB et al Bacteremic disseminated tuberculosis in sub-Saharan Africa: a prospective cohort study. Clin Infect Dis 2012; 55:242–50. 22511551 10.1093/cid/cis409 PMC3491770

[5] Crump JA, Wu X, Kendall MA et al Predictors and outcomes of *Mycobacterium tuberculosis* bacteremia among patients with HIV and tuberculosis co-infection enrolled in the ACTG A5221 STRIDE study. BMC Infect Dis 2015; 15:12. 25582793 10.1186/s12879-014-0735-5 PMC4297427

[6] Nakiyingi L, Ssengooba W, Nakanjako D et al Predictors and outcomes of mycobacteremia among HIV-infected smear-negative presumptive tuberculosis patients in Uganda. BMC Infect Dis 2015; 15:62. 25888317 10.1186/s12879-015-0812-4 PMC4332438

[7] Levi M, van der Poll T Inflammation and coagulation. Crit Care Med 2010; 38:S26–34. 20083910 10.1097/CCM.0b013e3181c98d21

[8] Opal SM, van der Poll T Endothelial barrier dysfunction in septic shock. J Intern Med 2015; 277:277–93. 25418337 10.1111/joim.12331

[9] Taylor FB Jr, Toh CH, Hoots WK, Wada H, Levi M Towards definition, clinical and laboratory criteria, and a scoring system for disseminated intravascular coagulation. Thromb Haemost 2001; 86:1327–30. 11816725

[10] Dhainaut JF, Yan SB, Joyce DE et al Treatment effects of drotrecogin alfa (activated) in patients with severe sepsis with or without overt disseminated intravascular coagulation. J Thromb Haemost 2004; 2:1924–33. 15550023 10.1111/j.1538-7836.2004.00955.x

[11] Fourrier F, Chopin C, Goudemand J et al Septic shock, multiple organ failure, and disseminated intravascular coagulation. Compared patterns of antithrombin III, protein C, and protein S deficiencies. Chest 1992; 101:816–23. 1531791 10.1378/chest.101.3.816

[12] Jong E, Louw S, van Gorp EC, Meijers JC, ten Cate H, Jacobson BF The effect of initiating combined antiretroviral therapy on endothelial cell activation and coagulation markers in South African HIV-infected individuals. Thromb Haemost 2010; 104:1228–34. 20886182 10.1160/TH10-04-0233

[13] Funderburg NT Markers of coagulation and inflammation often remain elevated in ART-treated HIV-infected patients. Curr Opin HIV AIDS 2014; 9:80–6. 24275673 10.1097/COH.0000000000000019 PMC3982388

[14] Graham SM, Rajwans N, Jaoko W et al Endothelial activation biomarkers increase after HIV-1 acquisition: plasma vascular cell adhesion molecule-1 predicts disease progression. AIDS 2013; 27:1803–13. 23807276 10.1097/QAD.0b013e328360e9fb PMC3883757

[15] Kuller LH, Tracy R, Belloso W et al Inflammatory and coagulation biomarkers and mortality in patients with HIV infection. PLoS Med 2008; 5:e203. 18942885 10.1371/journal.pmed.0050203 PMC2570418

[16] Schved JF, Gris JC, Arnaud A et al von Willebrand factor antigen, tissue-type plasminogen activator antigen, and risk of death in human immunodeficiency virus 1-related clinical disease: independent prognostic relevance of tissue-type plasminogen activator. J Lab Clin Med 1992; 120:411–9. 1517688

[17] Aukrust P, Bjornsen S, Lunden B et al Persistently elevated levels of von Willebrand factor antigen in HIV infection. Downregulation during highly active antiretroviral therapy. Thromb Haemost 2000; 84:183–7. 10959687

[18] Calza L, Pocaterra D, Pavoni M et al Plasma levels of VCAM-1, ICAM-1, E-selectin, and P-selectin in 99 HIV-positive patients versus 51 HIV-negative healthy controls. J Acquir Immune Defic Syndr 2009; 50:430–2. 19322038 10.1097/QAI.0b013e31819a292c

[19] Gattegno L, Bentata-Peyssare M, Gronowski S, Chaouche K, Ferriere F Elevated concentrations of circulating intercellular adhesion molecule 1 (ICAM-1) and of vascular cell adhesion molecule 1 (VCAM-1) in HIV-1 infection. Cell Adhes Commun 1995; 3:179–85. 8846020 10.3109/15419069509081285

[20] Kager LM, Blok DC, Lede IO et al Pulmonary tuberculosis induces a systemic hypercoagulable state. J Infect 2015; 70:324–34. 25455017 10.1016/j.jinf.2014.10.006

[21] Elkington PT, Ugarte-Gil CA, Friedland JS Matrix metalloproteinases in tuberculosis. Eur Respir J 2011; 38:456–64. 21659415 10.1183/09031936.00015411

[22] Hoheisel G, Sack U, Hui DS et al Occurrence of matrix metalloproteinases and tissue inhibitors of metalloproteinases in tuberculous pleuritis. Tuberculosis 2001; 81:203–9. 11466032 10.1054/tube.2000.0276

[23] Western Cape Government. National Antenatal Sentinel HIV Prevalence Survey. Western Cape, Cape Town, 2013 June 2014.

[24] Getahun H, Kittikraisak W, Heilig CM et al Development of a standardized screening rule for tuberculosis in people living with HIV in resource-constrained settings: individual participant data meta-analysis of observational studies. PLoS Med 2011; 8:e1000391. 21267059 10.1371/journal.pmed.1000391 PMC3022524

[25] National Health Laboratory Service, South Africa. https://labresults.nhls.ac.za/. Accessed 15 May 2016.

[26] Kostousov V, Fehr J, Bombeli T Novel, semi-automated, 60-min-assay to determine von Willebrand factor cleaving activity of ADAMTS-13. Thromb Res 2006; 118:723–31. 16430944 10.1016/j.thromres.2005.12.006

[27] Benjamini Y, Hochberg Y Controlling the false discovery rate: a practical and powerful approach to multiple testing. J R Stat Soc Series B Stat Methodol 1995; 57:289–300.

[28] Jacob ST, Pavlinac PB, Nakiyingi L et al *Mycobacterium tuberculosis* bacteremia in a cohort of HIV-infected patients hospitalized with severe sepsis in Uganda—high frequency, low clinical suspicion [corrected] and derivation of a clinical prediction score. PLoS One 2013; 8:e70305. 23940557 10.1371/journal.pone.0070305 PMC3734073

[29] Lewis DK, Peters RP, Schijffelen MJ et al Clinical indicators of mycobacteraemia in adults admitted to hospital in Blantyre, Malawi. Int J Tuberc Lung Dis 2002; 6:1067–74. 12546114

[30] Castoldi E, Hackeng TM Regulation of coagulation by protein S. Curr Opin Hematol 2008; 15:529–36. 18695379 10.1097/MOH.0b013e328309ec97

[31] Dahlback B C4b-binding protein: a forgotten factor in thrombosis and hemostasis. Semin Thromb Hemost 2011; 37:355–61. 21805441 10.1055/s-0031-1276584

[32] van der Meer JH, van der Poll T, van 't Veer C TAM receptors, Gas6, and protein S: roles in inflammation and hemostasis. Blood 2014; 123:2460–9. 24596417 10.1182/blood-2013-09-528752

[33] Huson MA, Kalkman R, Hoogendijk AJ et al Impact of HIV infection on the haemostatic response during sepsis and malaria. Br J Haematol 2016; 173:918–26. 26970408 10.1111/bjh.14006

[34] Claus RA, Bockmeyer CL, Sossdorf M, Losche W The balance between von-Willebrand factor and its cleaving protease ADAMTS13: biomarker in systemic inflammation and development of organ failure? Curr Mol Med 2010; 10:236–48. 20196724 10.2174/156652410790963367

[35] van Hinsbergh VW Endothelium—role in regulation of coagulation and inflammation. Semin Immunopathol 2012; 34:93–106. 21845431 10.1007/s00281-011-0285-5 PMC3233666

[36] De Groote MA, Nahid P, Jarlsberg L et al Elucidating novel serum biomarkers associated with pulmonary tuberculosis treatment. PLoS One 2013; 8:e61002. 23637781 10.1371/journal.pone.0061002 PMC3630118

[37] Walzl G, Ronacher K, Hanekom W, Scriba TJ, Zumla A Immunological biomarkers of tuberculosis. Nat Rev Immunol 2011; 11:343–54. 21475309 10.1038/nri2960

[38] Graham SM, Mwilu R, Liles WC Clinical utility of biomarkers of endothelial activation and coagulation for prognosis in HIV infection: a systematic review. Virulence 2013; 4:564–71. 23732995 10.4161/viru.25221 PMC5359730

[39] Al Wadi K, Ghatage P Efficacy of trebananib (AMG 386) in treating epithelial ovarian cancer. Expert Opin Pharmacother 2016; 17:853–60. 26933765 10.1517/14656566.2016.1161027

[40] Atkins MB, Gravis G, Drosik K et al Trebananib (AMG 386) in combination with sunitinib in patients with metastatic renal cell cancer: an open-label, multicenter, phase II study. J Clin Oncol 2015; 33:3431–8. 26304872 10.1200/JCO.2014.60.6012

[41] Chiu JW, Hotte SJ, Kollmannsberger CK et al A phase I trial of ANG1/2-Tie2 inhibitor trebaninib (AMG386) and temsirolimus in advanced solid tumors (PJC008/NCImusical sharp9041). Invest New Drugs 2016; 34:104–11. 26686201 10.1007/s10637-015-0313-8 PMC4718956

[42] Iglesias D, Alegre J, Aleman C et al Metalloproteinases and tissue inhibitors of metalloproteinases in exudative pleural effusions. Eur Respir J 2005; 25:104–9. 15640330 10.1183/09031936.04.00010504

[43] Hasibuan FM, Shiratori B, Senoputra MA et al Evaluation of matricellular proteins in systemic and local immune response to *Mycobacterium tuberculosis* infection. Microbiol Immunol 2015; 59:623–32. 26337438 10.1111/1348-0421.12320

[44] Coussens LM, Fingleton B, Matrisian LM Matrix metalloproteinase inhibitors and cancer: trials and tribulations. Science 2002; 295:2387–92. 11923519 10.1126/science.1067100

[45] Walker NF, Clark SO, Oni T et al Doxycycline and HIV infection suppress tuberculosis-induced matrix metalloproteinases. Am J Resp Crit Care 2012; 185:989–97. 10.1164/rccm.201110-1769OC PMC3359940 22345579

[46] Brenchley JM, Price DA, Schacker TW et al Microbial translocation is a cause of systemic immune activation in chronic HIV infection. Nat Med 2006; 12:1365–71. 17115046 10.1038/nm1511

[47] Marchetti G, Tincati C, Silvestri G Microbial translocation in the pathogenesis of HIV infection and AIDS. Clin Microbiol Rev 2013; 26:2–18. 23297256 10.1128/CMR.00050-12 PMC3553668

[48] Zink AR, Sola C, Reischl U et al Characterization of *Mycobacterium tuberculosis* complex DNAs from Egyptian mummies by spoligotyping. J Clin Microbiol 2003; 41:359–67. 12517873 10.1128/JCM.41.1.359-367.2003 PMC149558

[49] Feruglio SL, Troseid M, Damas JK, Kvale D, Dyrhol-Riise AM Soluble markers of the Toll-like receptor 4 pathway differentiate between active and latent tuberculosis and are associated with treatment responses. PLoS One 2013; 8:e69896. 23875007 10.1371/journal.pone.0069896 PMC3713063

[50] Subbarao S, Wilkinson KA, van Halsema CL et al Raised venous lactate and markers of intestinal translocation are associated with mortality among in-patients with HIV-associated TB in rural South Africa. J Acquir Immune Defic Syndr 2015; 70:406–13. 26186506 10.1097/QAI.0000000000000763 PMC4625603

