# Supplementary material for: Hemostatic Changes Associated With Increased Mortality Rates in Hospitalized Patients With HIV-Associated Tuberculosis: A Prospective Cohort Study
Source: J Infect Dis. 2016 Nov 7;215(2):247–58. doi: 10.1093/infdis/jiw532 (PMC5439596; doi:10.1093/infdis/jiw532)
Supplement: Supplementary Data [file jiw532_Supplementary_Data.zip › jiw532supp_table1.docx]

**Supplementary Table Scoring system for disseminated intravascular coagulation**

| **Score** | **0** | **1** | **2** |
| --- | --- | --- | --- |
| Platelet count | > 100*10^9^/L | < 100*10^9^/L | < 50*10^9^/L |
| Elevated D-dimer | < 0.5 µg/mL | 0.5-8.2 µg/mL | > 8.2 µg/mL |
| Prolonged prothrombin time | < 3 seconds | 3-6 seconds | > 6 seconds |
| Fibrinogen level | > 1.0 g/L | < 1.0 g/L |  |
| **Fulfils criteria if total score ≥ 5** |  |  |  |

Table showing the scoring system for disseminated intravascular coagulation (DIC) adapted from Taylor et al, 2001^15^, based on reference values for D-dimer in our laboratory. Patients scoring a total of 5 points or higher were regarded as fulfilling criteria for DIC.
